# Bobbin Tool Friction Stir Welding of Aluminum: Parameters Optimization Using Taguchi Experimental Design

**DOI:** 10.3390/ma15082771

**Published:** 2022-04-09

**Authors:** Mohamed M. Z. Ahmed, Kamel Touileb, Mohamed M. El-Sayed Seleman, Ibrahim Albaijan, Mohamed I. A. Habba

**Affiliations:** 1 Mechanical Engineering Department, College of Engineering at Al Kharj, Prince Sattam Bin Abdulaziz University, Al Kharj 16273, Saudi Arabia; k.touileb@psau.edu.sa (K.T.); i.albaijan@psau.edu.sa (I.A.); 2 Department of Metallurgical and Materials Engineering, Faculty of Petroleum and Mining Engineering, Suez University, Suez 43512, Egypt; mohamed.elnagar@suezuniv.edu.eg; 3 Mechanical Department, Faculty of Technology and Education, Suez University, Suez 43518, Egypt; mohamed.atia@suezuniv.edu.eg

**Keywords:** friction stir welding, AA1050, bobbin tool, mechanical properties, Taguchi design, ANOVA, regression model

## Abstract

This work aims to optimize the performance evaluation characteristics such as the temperature at the weld center of the lap joint (Tw), the tensile shear load (TSL), and the hardness using an experimental design experiment for bobbin tool friction stir welding (BT-FSW) of AA1050 lap joints. BT-FSW is characterized by a fully penetrated pin and double-sided shoulder that promote symmetrical solid-state welds. This study contributes to improving the quality of 10 mm thick lap joints and addressing challenges to obtaining a sound weld deprived of any defects. Taguchi L9 orthogonal array (OA) experimental design was performed. Three different pin shapes (cylindrical, triangular, and square) and three levels of welding travel speeds of 200, 400, and 600 mm/min were selected as input controllable process parameters at a constant tool rotation speed of 600 rpm. A travel speed of 200 mm/min with square pin geometry significantly improves the TSL of the joint up to 6491 N. However, the hardness characteristic is optimized by using 600 mm/min travel speed and a cylindrical tool pin. The minimum temperature in the weld joint can be obtained using 600 mm/min or more with triangular pin geometry. From ANOVA results, it was seen that the BT-FSW of AA 1050 thick lap joints performance in terms of TLS and Tw were greatly influenced by travel speed; however, the tool shape influences the hardness more. For the validation of the models, BT-FSW experiments have been carried out for AA1050 using the applied processing parameters. Furthermore, regression models were developed to predict the Tw, TSL, and hardness. The calculated performance properties from the mathematical models were in an acceptable range compared to the measured experimental values.

## 1. Introduction

Aluminum and its alloys have been vastly used in many industrial applications such as transportation, marine, and aerospace for their high strength and light weight [1,2]. Welding based fusion techniques are widely used in the joining of aluminum alloys [3,4,5]. On the contrary, these techniques have some problems like solidification cracking, porosity, and stress corrosion cracking [6,7]. Friction stir welding (FSW) is a solid-state joining process used for welding metals, especially aluminum and its alloys [8,9,10]. During a conventional friction stir welding (CFSW) using a tool consisting of a pin and one shoulder, a backing plate is required to support the joint plates, which leads to some limitations in the welding process. In addition, not being able to weld hollow sections structures may result in root defects during the FSW if the plunging depth of the conventional tool is incorrectly selected or a shortened pin length is utilized. These root defects are commonly very difficult to reveal [11,12,13]. A bobbin tool (BT) is an innovative FSW tool design. The bobbin tool friction stir welding (BTFSW) is an effective technique to outdo the limitations of the CFSW [14,15]. In this technique, the BT has two shoulders attached with the joint plates, one above (upper shoulder) and one below (lower shoulder) the joint plates, and the tool pin connects the two shoulders and penetrates through the joint thickness. The lower shoulder of the BT replaces the backing plate used in CFSW; because of this, the BTFSW of the hollow section structure can be welded [9]. Furthermore, the welding of joint plates on both sides in one pass completely eliminates the root defect. Until now, aluminum series, such as 1xxx [16,17], 2xxx [18,19], 6xxx [19,20], and 7xxx [21,22], have been successfully welded using the BTFSW technique in previous investigations. For instance, AA1050 alloy was successfully welded in a 10 mm lap joint using BT design by Ahmed et al. [9]. They used three different pin geometries (cylindrical, triangle, and square) to produce AA1050 lap joints at a constant rotation speed of 600 rpm and a wide range of travel speeds from 50 to 1000 mm/min. They reported that the pin geometry and the welding travel speed significantly affected the welding temperature, hardness, and tensile strength of the welded joints. Zhang et al. [14] experimentally studied the BTFSW of 6 mm 2A14-T6 aluminum alloy using a cylindrical pin with three flat surfaces at a constant rotation speed of 400 rpm, and different travel speeds of 50, 100, and 150 mm/min. They concluded that the all-welded joints have a homogeneity hardness profile through the thickness of the welded joints. The tensile strength of the welded joints increased with increasing travel speed, and a maximum tensile strength was obtained at 150 mm/min with 75% joint efficiency. Actually, the researchers used the experimental methods to investigate the impact of the process parameters, changing one parameter at a time while keeping the other variables constant. This research technique based on only an experimental approach is time and resource-intensive. Taguchi statistical design is a helpful approach to identify influential factors from many variables and then reducing the number of experiments. The Taguchi approach has been involved in many manufacturing techniques such as casting [23,24,25], fusion welding [26,27,28,29], and powder metallurgy [30,31,32]. In the literature, some studies applied the Taguchi method in the CFSW to optimize the CFSW parameters [33,34,35,36]. Moreover, no studies were found applying the Taguchi method to optimize the BTFSW parameters. Thus, the current research focused on the Taguchi statistical design to optimize and analyze the experiments for the BTFSW of the 10 mm AA1050 thick lap joints welded using three different pin geometries at different travel speeds of 200, 400, and 600 mm/min at a constant rotation speed of 600 rpm.

## 2. Materials and Methods

**Figure 1:**
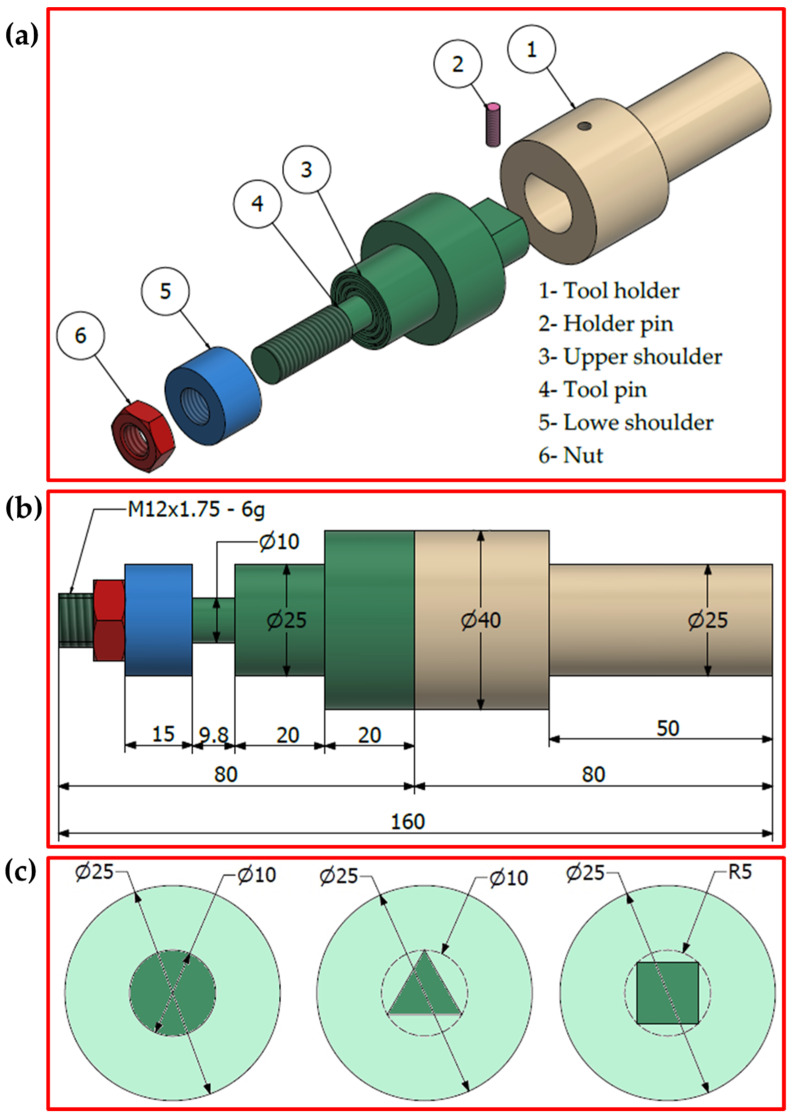
(**a**) Exploded 3D drawings, (**b**) dimensions, and (**c**) schematic drawings of bobbin tool [9]. (unit: mm).

**Figure 2:**
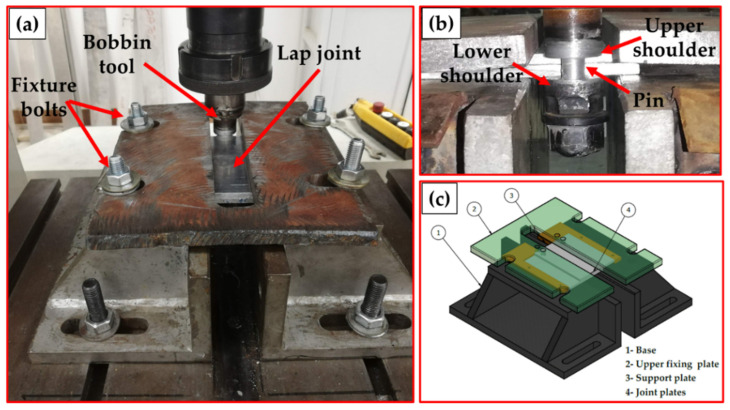
(**a**) Front photo and (**b**) back photo of BT-FSW of AA1050 thick lap joint. (**c**) 3D drawing of the used fixture system for BT-FSW process [9].

Three different bobbin tool pin geometries of cylindrical, square, and rectangular cross sections with the bobbin tool were used in the current study. Dimensions and descriptions of the BT-FSW tools are illustrated in Figure 1 and Table 1. The range of BT-FSW parameters examined was a constant rotation speed of 600 rpm and different welding travel speeds of 200, 400, and 600 mm/min. For this purpose, three bobbin tools were manufactured from H13 tool steel and used to conduct the BT-FSW experiments for the AA1050-H14 plates of 10 mm thickness in lap joint configuration. A fixing system was designed and manufactured for BT-FSW purposes. Figure 2a,b shows the BT-FSW of 10 mm AA1050-H14 lap joints using this fixture system, and Figure 2c shows the 3D design drawing of the BT-FSW fixing system with all components identified. The generated temperatures in the NG, AS, and RS upon the BT-FSW were recorded at the surface just behind the tool using an infrared thermometer (Quicktemp 860-T3, Testo Company—Berlin, Germany). A special setup was created using Modern Digital Multimeter (MDM) model (UT61B—Zhejiang, China) with thermocouple type “K” in order to measure the temperature at the AS and RS. With an MDM device, two thermocouples were used to collect the temperatures by placing them in two holes close to the weld pass in the heat-affected zone (HAZ) with 3 mm diameters and 3 mm depth drilled on both the AS and RS [9].

**Figure 3:**
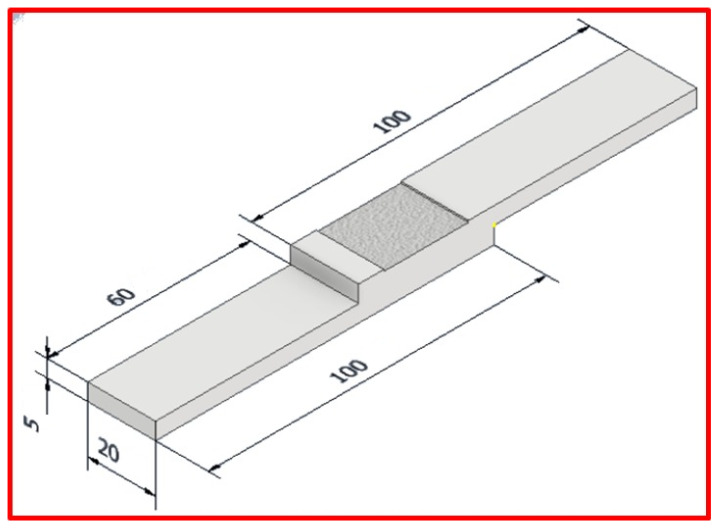
Dimensions of tensile shear lap joints. (Dimensions are in mm).

Furthermore, the specimens of the tensile-shear lap (TSL) test were cut along the transverse directions of the joint. Figure 3 represents the dimensions of TSL specimens. The TSL test was carried out using Instron universal test machine model, Instron-4208 (Norwood, MA, USA). Finally, the hardness measurements of the welded joints were done using the Vickers hardness machine model, HWDV-75 (TTS Unlimited company, Osaka, Japan), using a load of 0.5 kg for 15 sec dwell time. The chemical composition and the mechanical properties of the investigated AA1050-H14 are presented in Table 2.

The BTFSW process parameters that may influence the quality of the FSW joints are travel speed and tool pin geometry. The rotational speed is maintained constant during this study at 600 rpm. In the present investigation, three levels of these process parameters were considered after conducting trial runs. The FSW process parameters and their levels are given in Table 3.

## 3. Results and Discussion

### 3.1. Taguchi Method

Design of experiments (DoE) is a statistical procedure for planning experiments so that the gathered data can be analyzed to find out the best optimal parameters without increasing experimental time and cost, and without compromising the quality and reliability of a certain process. The Taguchi method is a DoE variant developed by Genichi Taguchi, with a special design of orthogonal arrays that provide the optimum parameters settings. In this work, data analysis based on the Taguchi method (L9 orthogonal array) is performed by utilizing the Minitab statistical software (version 17, Minitab, Pennsylvania State University, State College, PA, USA) to estimate the significant factors of the 10 mm AA1050 BT-FSW thick lap joints and main effects using limited experimental tests only. In this work, the input parameters are travel speed and tool pin shape, as mentioned in Table 4. The responses such as lap tensile shear (TSL), hardness, and the temperature attained in the weld zone (WZ) are the output parameters. To evaluate the responses, the category quality of the larger the better is chosen to calculate the S/N ratio for the TSL and hardness Equation (1). However, the lower the better is recommended to evaluate the temperature attained in the WZ of the produced joints. Hence, Equation (2) has been used to calculate the Signal to noise ratio (S/N ratio):

(1) $$S/N\;\mathrm{ratio}\;\mathrm{for}\;\mathrm{the}\;\mathrm{larger}\;\mathrm{the}\;\mathrm{better}=-10\;\log\;\frac{1}{n}\sum 1/y_{i}^{2}$$

(2) $$S/N\;\mathrm{ratio}\;\mathrm{for}\;\mathrm{the}\;\mathrm{smaller}\;\mathrm{the}\;\mathrm{better}=-10\;\log\;\frac{1}{n}\sum^{\;}y_{i}^{2}$$

*n*: is the number of experiments of the orthogonal design, which for this case is *n* = 9, *y*_i_: is the response at each experiment.

### 3.2. Effect of Process Parameters on BT-FSW of AA1050 Thick Lap Joint

#### 3.2.1. Effect of Process Parameters on the Temperature at Weld Center (Tw)

**Figure 4:**
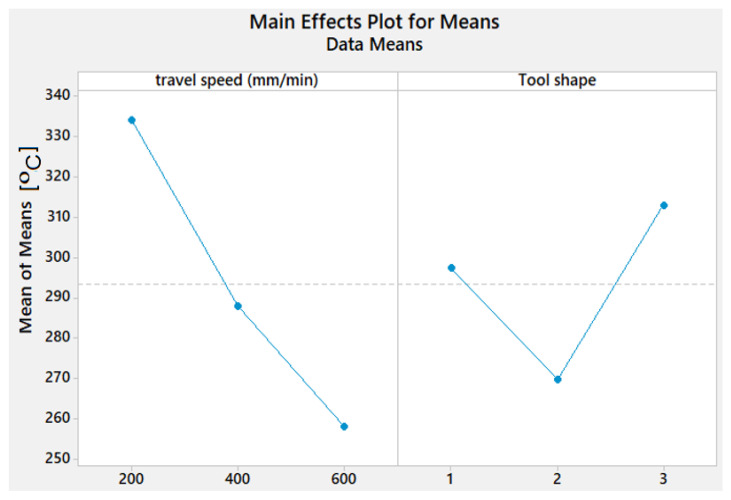
Effect of process parameters on the Tw of AA1050 thick lap joints.

Figure 4 depicts the effect of process parameters on Tw of the stir zone. It can be noted that Tw decreases with a rise in travel speed. It is also observed from Figure 4 that the Sq pin geometry shows the highest Tw value. By using the Tr pin shape, we get the lowest Tw.

#### 3.2.2. Effect of Process Parameters on the TSL

**Figure 5:**
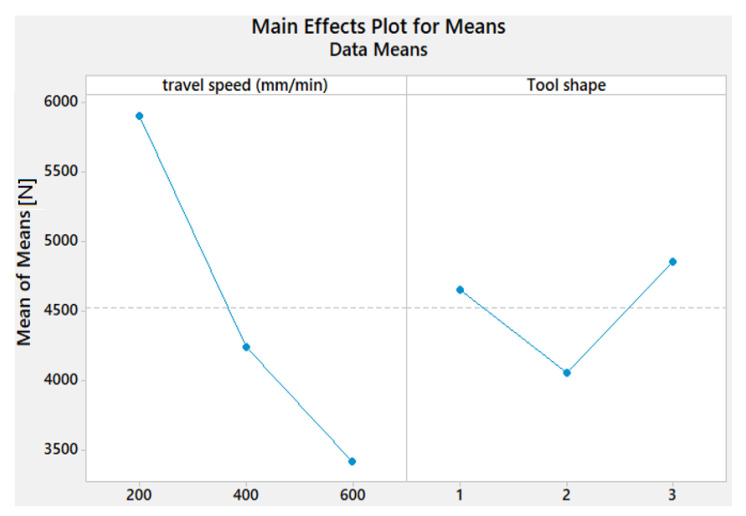
Effect of process parameters on the TSL of AA1050 thick lap joints.

Figure 5 shows that TSL decreases as the travel speed increases. The maximum value of TSL is obtained with Sq tool shape, while the lowest value is attained with the Tr pin shape.

#### 3.2.3. Effect of Process Parameters on Hardness

**Figure 6:**
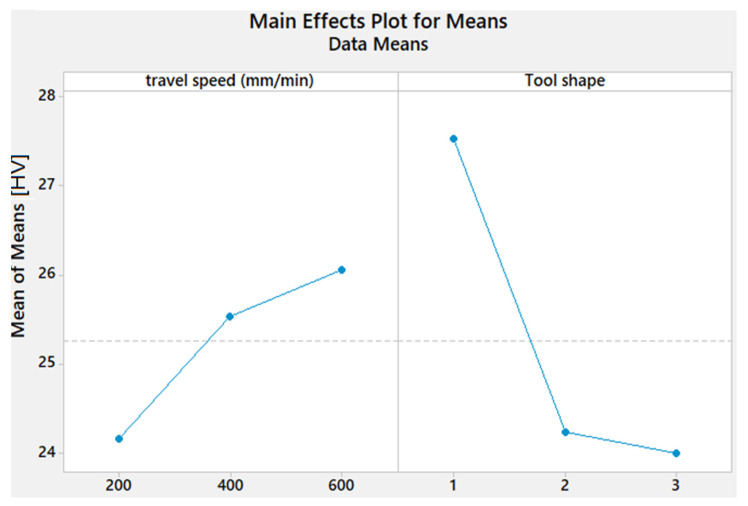
Effect of process parameters on the hardness of AA1050 thick lap joints.

Figure 6 depicts the effect of process parameters on the hardness in the WZ. The hardness increases as the travel speed increases. The maximum mean value of the hardness is obtained with Cy pin shape, and the lowest value is observed with the Sq pin shape.

### 3.3. Selection of Optimum FSW Lap Joint Conditions for Tw, TSL and Hardness

S/N ratio response is needed to find out accurately the optimal combinations of the parameter levels. The obtained S/N ratio response for the Tw is shown in Table 5, displaying the mean S/N ratio graph obtained by the Minitab software. A higher S/N ratio represents the minimum variation difference between the desirable output and measured output.

**Figure 7:**
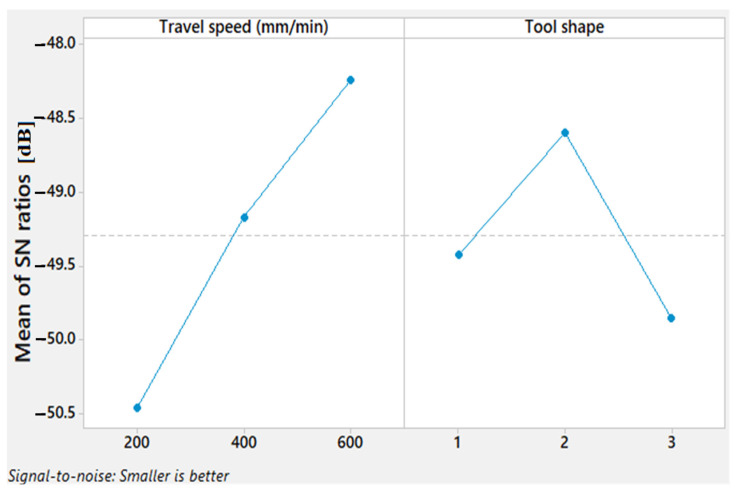
Mean S/N ratio for Tw.

It can be noticed that the highest mean S/N ratio obtained for Tw are travel speed at 600 mm/min and Tr tool shape, as shown in Figure 7.

**Figure 8:**
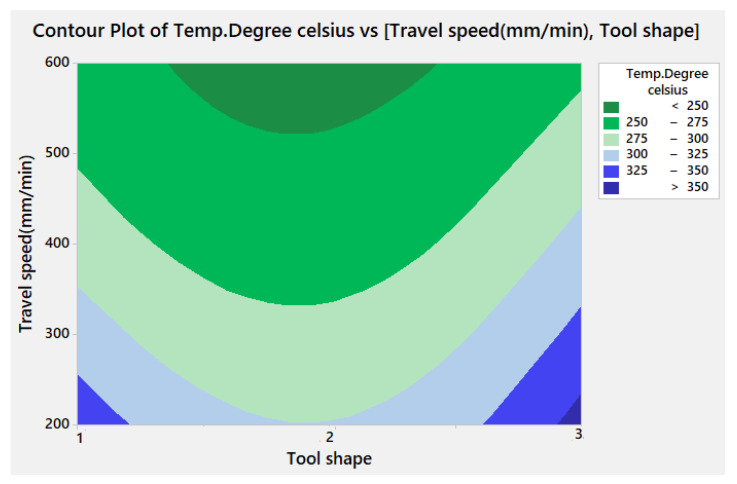
Contour plot for Tw against travel speed and tool shape.

Figure 8 represents the contour plot for Tw as a function of travel speed and tool shape. It reveals that the temperature can decrease significantly if we use a travel speed of more than 600 mm/min while maintaining the same type of pin tool shape.

The results of the S/N ratio response for TSL displayed in Table 6 show that the 200 mm/min travel speed gave the highest mean S/N ratio and the Sq pin shape exhibits the highest S/N in comparison to the other pin geometries.

**Figure 9:**
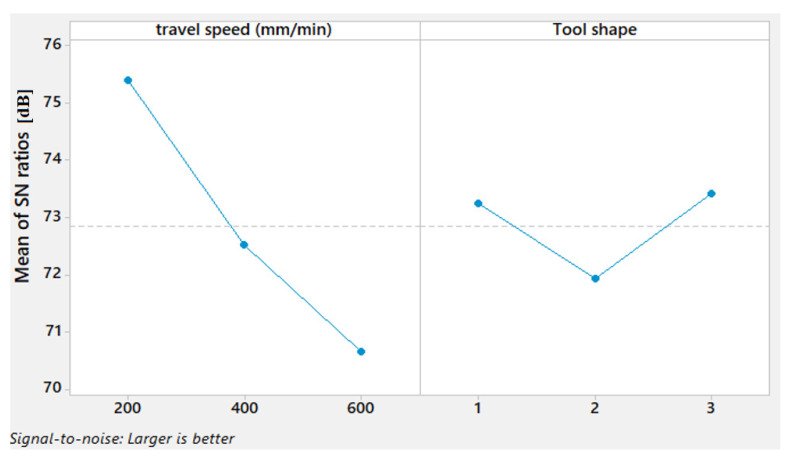
Mean S/N ratio for TSL of AA1050 thick lap joints.

The mean S/N ratio graph (Figure 9) reveals that the higher difference range for travel speed between the highest and the lowest of S/N values is remarked compared to the difference range for the tool shapes. This indicates the importance of the travel speed in influencing the TSL property.

**Figure 10:**
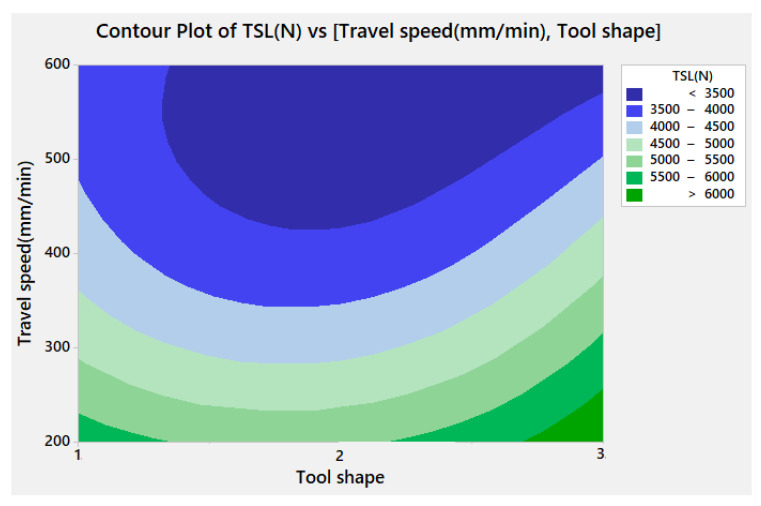
Contour plot for TSL versus travel speed and tool shape.

Figure 10 illustrates the contour plot for TSL versus the travel speed and the tool shape. It can be remarked that the maximum TSL can be obtained only with the Sq tool shape coupled with 200 mm/min travel speed.

**Figure 11:**
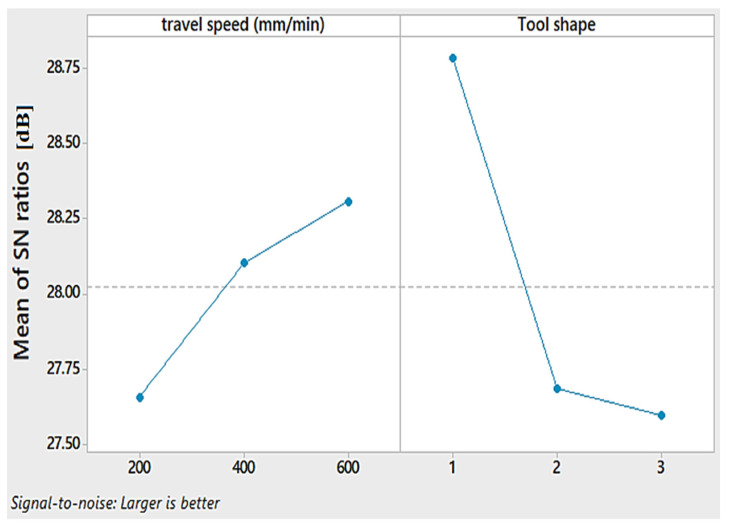
Mean S/N ratio for the hardness of AA1050 thick lap joints.

The obtained S/N ratio for the hardness is shown in Table 7 and Figure 11. A higher S/N ratio for hardness is achieved with a travel speed of 600 mm/min and Cy pin shape, respectively.

**Figure 12:**
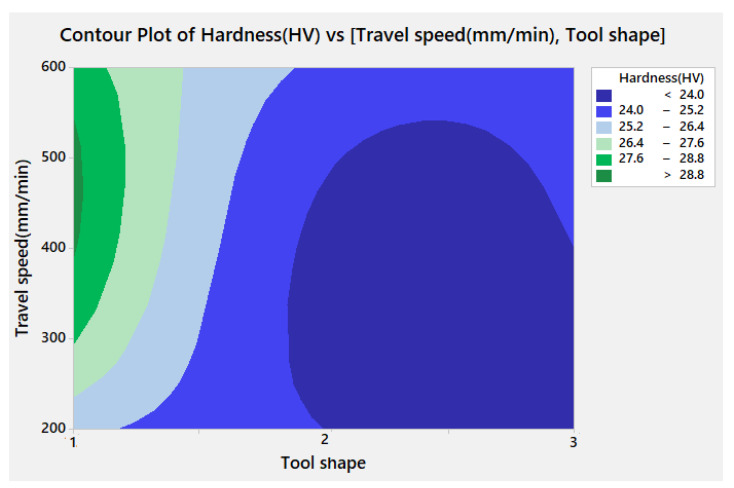
Contour plot for hardness against travel speed and tool shape.

Figure 12 represents the contour plot for hardness versus travel speed and tool shape, where it is clearly shown that the hardness can be increased while using the travel speed in the range between 400 and 500 mm/min with Cy tool pin shape.

### 3.4. ANOVA for Tw, TSL and Hardness

ANOVA helps informally test the significance of all main factors. The purpose of ANOVA is to find out the significant factor in the process. The ANOVA for the S/N ratio is collected and listed in Table 8, Table 9 and Table 10. The S/N ratio at each level for a parameter is calculated. The difference between the maximum and minimum of the S/N ratio in each parameter verified the effectiveness of the parameter in the process.

To determine the relative effect of the welding parameters, the standard ANOVA procedure was performed using the mean values. Table 8 indicates the greater contribution of the welding parameters to minimize the temperature in the weld zone in order to avoid grain coarsening of the friction stir welded joints. The most significant parameter is travel speed, with a contribution of 73.64%. This result is in good agreement with the response table for S/N ratios (Table 5).

Table 9 shows the ANOVA table indicating the greater importance of the welding parameters to maximize the TSL of the friction stir welded joints. The most significant parameter is travel speed, with a contribution of 84.77%. This result is in good agreement with the response table for S/N ratios (Table 6).

Table 10 shows the ANOVA results indicating the greater influence of the welding parameters to maximize the hardness of the friction stir welded joints. The most significant parameter is tool geometry, with a contribution of 71.51%. This result is in good agreement with the response table for S/N ratios (Table 7).

### 3.5. Confirmation Test

For validating the Taguchi predicted optimum conditions, confirmation tests need to be performed.

At the Taguchi predicted optimum BT-FSW thick lap joint conditions, the confirmation experiments were performed, and the results were shown in Table 11, Table 12 and Table 13 for Tw, TSL, and hardness, respectively. The predicted optimum FSW lap joint conditions for Tw, TSL, and hardness give an improvement in the performance characteristic results. From Table 11, Table 12 and Table 13, it was observed that S/N ratios of predicted and optimal FSW lap joint conditions are very close for both Ra and Vb defined earlier.

The S/N ratio improvement found at the optimal cutting condition for Tw, TSL, and hardness were: 47.75 dB, 76.25 dB, and 29.01 dB, respectively, when compared to initial parameter settings as shown in Table 11, Table 12 and Table 13. From the Taguchi predicted optimum FSW lap joint conditions, Tw reduction was achieved to be 18.85% and obvious increasing of TSL and hardness about 25.94% and 16.07%, respectively, when compared to initial parameter conditions.

#### 3.5.1. Tw (Center Weld Temperature)

From the confirmation experiments, it was found that the temperature of an FSW lap joint gives a favorable result over the initial parameter conditions as shown in Table 11. The confirmation test decreased to 244 °C against the initial conditions of 290 °C which is very favorable for the soundness of a joint.

The experiments using triangular pin geometry and two different travel speeds of 800 mm/min and 1000 mm/min were conducted to verify and confirm the predictions mentioned above. The temperature attained in the lap joint were decreased to 251 °C and 219 °C, respectively.

#### 3.5.2. TSL (Tensile Shear Load)

From the confirmation experiments, it was found that the TSL of the BT-FSW thick lap joint gives favorable results over the initial parameter conditions, as shown in Table 12. The confirmation test for TSL reached 6491 N against the initial condition 4807 N. The joint weld carried out with the optimal condition withstands more from the external load, which meets the industry’s needs.

#### 3.5.3. Hardness

It was found that the hardness of the BT-FSW thick lap joint gives favorable results over the initial parameter conditions, as shown in Table 13. The confirmation test for hardness attained 28.20 HV against the initial conditions of 23.7 HV.

The experiments using cylindrical pin geometry and two different travel speeds of 450 mm/min and 500 mm/min were conducted to verify and confirm the predictions mentioned above. The hardness reached in the lap joint was 28.8 HV for a travel speed of 450 mm/min. Moreover, the hardness is maximized and attained 32.8 HV while using a travel speed of 500 mm/min.

### 3.6. Modeling

In the present work, linear regression analysis in Minitab statistical software V17 has been used to develop the predictive mathematical models for the dependent variables of Tw, *TSL*, and hardness as a function of travel speed (*Tr. speed*) and tool shape (*T. shape*), respectively. No transformation has been performed on each response. The predictive equations obtained from the regression are:

(3) $$Tempreture\;\left(Degree\;Celsius\right)=354.2-0.1900\;Tr.\;speed\;\left(\frac{mm}{min}\right)+7.67\;T.shape.$$

$\mathrm{with}\;R^{2}=75.43\%$

(4) $$TSL\;\left(N\right)=6806-6.20\;Tr.\;speed\;\left(\frac{mm}{min}\right)+101\;T.\;shape.$$

$\mathrm{with}\;R^{2}=83.19\%$

(5) $$Hardness\;\left(HV\right)=26.89+0.00475\;Tr.\;speed\;\left(\frac{mm}{min}\right)-1.767\;T.\;shape$$

$\mathrm{with}\;R^{2}=73.60\%$

The goodness of fit is measured by several statistical parameters, including the coefficient of determination (*R*^2^). The capability of developed models was checked using a coefficient of determination *R*^2^. This coefficient may vary between 0% (bad fit) and 100% (ideal fit). However, there is no clear rule to classify whether an *R*^2^ value is high or low since it depends on the domain and it is case-sensitive even in the same domain. Different scholars have different opinions on what constitutes a good value for this performance indicator, according to those two scholars. In the present study, the developed regression models for Tw, *TSL*, and hardness have high *R*^2^ values as 75.43%, 83.19%, and 73.60%, respectively. We can consider that our three *R*^2^ (minimum *R*^2^ = 73.60%) have a substantial value or even good values since we are obtaining this performance with a relatively limited number of data (9 experiments).

**Figure 13:**
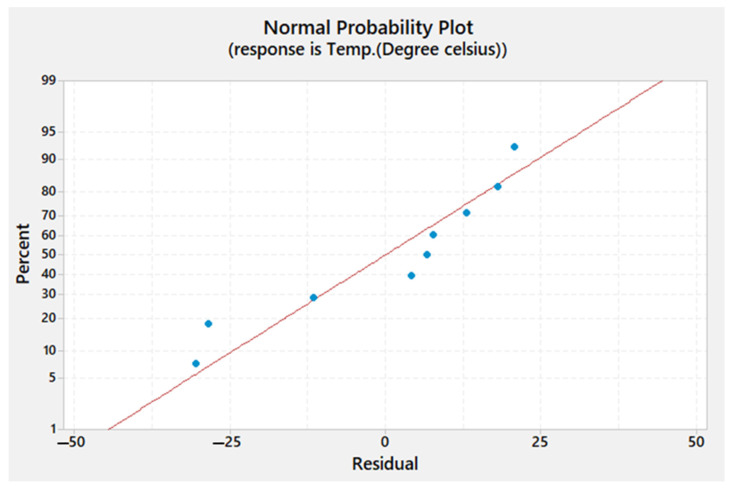
Normal probability plot of the residuals for the temperature of the weld zone.

**Figure 14:**
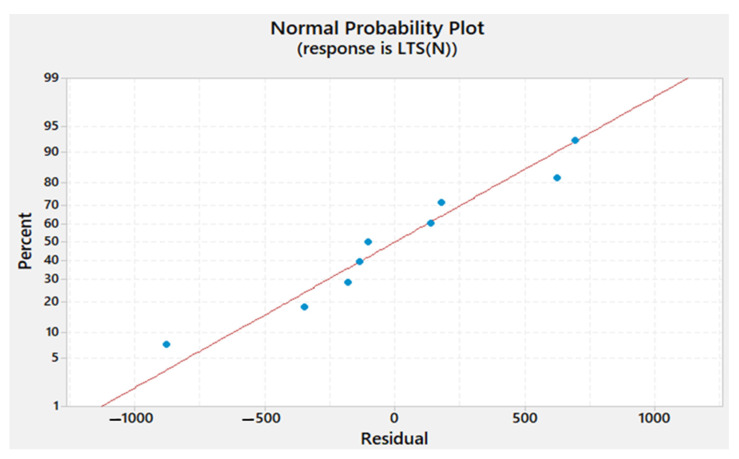
Normal probability plot of the residuals for the TSL.

**Figure 15:**
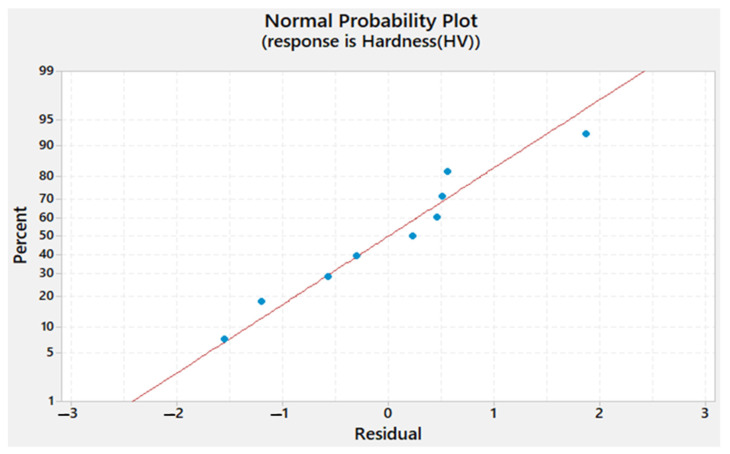
Normal probability plot of the residuals for the hardness.

The residual plots obtained for Tw, *TSL*, and hardness are shown in Figure 13, Figure 14 and Figure 15, respectively. It can be seen that the residuals fall near the straight line for Tw, *TSL*, and hardness, which infers that the developed model coefficient models are weighty.

Validation tests were carried out to confirm the reliability and accuracy of the developed models, and results are shown in Table 14. The testing results were taken randomly from the L9 orthogonal experimental design. The conformation results found that predicted results from the models and experimental results were in very acceptable agreement within the given range of parameters.

## 4. Conclusions

In this work, optimization using Taguchi experimental design for the BT-FSW of AA1050 thick lap joints was carried out. The following main conclusions can be drawn:

1. Optimum BT-FSW thick lap joint condition combination for obtaining the low temperature at weld center was found at a travel speed of 600 mm/min and Tr pin shape. It was observed that an 18.85% reduction of temperature at weld center was found at the Taguchi determined optimum condition.
2. The *TSL* is maximized at optimum welding conditions, which is the travel speed of 200 mm/min using the Sq pin geometry. The Taguchi determined optimum lap joint conditions increased the *TSL* by 31.18%.
3. The hardness property is maximized at the optimum welding condition, which is 600 mm/min travel speed using the Cy pin geometry. The Taguchi determined optimum lap joint conditions increased the TSL by 16.07%.
4. From the ANOVA, it was observed that Tw and TSL were significantly affected by the travel speed with a contribution of 73.64% and 84.77%, respectively. However, the hardness is mostly influenced by the tool shape, with a contribution of 71.51%.
5. The mathematical regression models for Tw, *TSL*, and hardness are in acceptable concordance with the experimental results. The predicted response results and experimental results are in good conformity.

## Figures and Tables

**Table 1:** Bobbin tool friction stir welding (BT-FSW) tools’ specifications [9].

Description of BT-FSW Tool	
Upper and lower shoulder	25 mm diameter
Shoulder surface	Concave (≈6°) and cavities
Pin dimensions	Cylindrical with10 mm diameter
	Square pin with inside circle diameter 10 mm
	Triangle pin with inside circle diameter 10 mm
Pin profile	Smooth
Shoulder gap	9.5 mm

**Table 2:** Nominal chemical composition and mechanical properties of aluminum alloy AA1050-H14.

Elements in wt.%						
Si	Fe	Cu	Mg	Zn	Ti	Al
0.07	0.26	<0.001	<0.001	<0.002	<0.007	Bal.
Yield strength, MPa		Tensile strength, MPa			Hardness, HV	
60		100			30	

**Table 3:** Process parameters and their levels.

Process Parameters	Units	Levels		
		1	2	3
Travel speed	mm/min	200	400	600
Tool shape	-	Cylindrical (Cy)	Triangular (Tr)	Square (Sq)

**Table 4:** Experimental plan, experimental results, and their calculated S/N ratios.

Experiment Runs	Controllable Input Parameters		Experimental Results			S/N Ratios of Results		
			Temp. °C	TSL (N)	Hardness (HV)	Temp. (dB)	TSL (dB)	Hardness (dB)
	Travel Speed (mm/min)	Tool Shape						
1	200	Cy	342	5806	25.50	−50.68	75.28	28.13
2	200	Tr	301	5419	24.00	−49.57	74.68	27.60
3	200	Sq	360	6491	23.00	−51.12	76.25	27.23
4	400	Cy	290	4292	28.90	−49.25	72.65	29.22
5	400	Tr	265	3648	23.70	−48.46	71.24	27.50
6	400	Sq	309	4807	24.00	−49.80	73.64	27.60
7	600	Cy	261	3881	28.20	−48.33	71.78	29.01
8	600	Tr	244	3108	25.00	−47.75	69.85	27.96
9	600	Sq	270	3285	25.00	−48.63	70.33	27.96

**Table 5:** Mean S/N ratio response table for Tw.

Process Parameters	Mean of S/N				
	Level 1	Level 2	Level 3	Max-Min	Rank
Travel speed	−50.46	−49.17	−48.24	2.2	1
Tool shape	−49.42	−48.59	−49.85	1.26	2

**Table 6:** Mean S/N ratio response table for TSL.

Process Parameters	Mean of S/N				
	Level 1	Level 2	Level 3	Max-Min	Rank
Travel speed (mm/min)	75.40	72.51	70.65	4.75	1
Tool shape	73.24	71.92	73.40	1.48	2

**Table 7:** Mean S/N ratio response table for hardness.

Process Parameters	Mean of S/N				
	Level 1	Level 2	Level 3	Max-Min	Rank
Travel speed (mm/min)	27.66	28.11	28.31	0.65	2
Tool shape	28.78	27.69	27.60	1.19	1

**Table 8:** ANOVA for Tw.

Parameter	Degree of Freedom	Sum of Squares	Mean Squares	Contribution (%)
Travel speed mm/min	2	8802.9	4401.44	73.64
Tool shape	2	2849.6	1424.78	23.84
Residual error	4	301.8	75.44	2.52
Total	8	11,954.2		

**Table 9:** ANOVA for TSL.

Parameter	Degree of Freedom	Sum of Squares	Mean Squares	Contribution (%)
Travel speed mm/min	2	34.343	17.1717	84.77
Tool shape	2	3.949	1.9746	9.75
Residual error	4	2.221	0.5553	5.48
Total	8	40.514		

**Table 10:** ANOVA for Hardness.

Parameter	Degree of Freedom	Sum of Squares	Mean Squares	Contribution (%)
Travel speed mm/min	2	0.6663	0.33315	18.18
Tool shape	2	2.6196	1.30978	71.51
Residual error	4	0.3774	0.09436	10.3
Total	8	3.6633		

**Table 11:** Confirmation test results for center weld temperature.

	Initial Process Parameter	Optimal Process Parameter	
		Prediction	Experiment
Level	400.1	600.2 (Tr)	600.2 (Tr)
Tw (°C)	290	235	244
S/N ratio (dB)	−49.25	−47.5420	− 47.75
Percentage reduction of Tw 18.85%			

**Table 12:** Confirmation test results for TSL.

	Initial Process Parameter	Optimal Process Parameter	
		Prediction	Experiment
Level	400.1	200.3 (Sq)	200.3 (Sq)
TSL(N)	4807	6240	6491
S/N ratio (dB)	72.65	75.95	76.25
Percentage increase of Tw 25.94%			

**Table 13:** Confirmation test results for hardness.

	Initial Process Parameter	Optimal Process Parameter	
		Prediction	Experiment
Level	400.2	600.1 (Cy)	600.1 (Cy)
Hardness (HV)	23.7	28.34	28.20
S/N ratio (dB)	27.50	29.07	29.01
Percentage increase of hardness 16.07%			

**Table 14:** The soundness of the developed models.

Run	Experimental			Predicted			Discrepancy between Models and Experiments (%)		
	Tw °C	*TSL* (N)	Hardness (HV)	Tw °C	*TSL* (N)	Hardness (HV)	Tw	*TSL*	Hardness
1	342	5806	25.5	315	5703	26.1	7.9	1.77	2.24
2	301	5419	24	332	5768	24.3	10.3	6.44	1.27
3	360	6491	23	339	5869	22.5	5.8	9.6	2
4	290	4292	28.9	277	4427	27	4.5	3.1	6.49
8	244	3108	25	256	3288	26.2	5	5.79	4.83
9	270	3285	25	263	3389	24.44	2.6	3.19	2.24

## Data Availability

Data will be available upon request through the corresponding author.
